# Metabolic reprogramming and immunosenescence in colorectal cancer: mechanisms and therapeutic implications

**DOI:** 10.3389/fcell.2025.1662464

**Published:** 2025-08-13

**Authors:** Jun Zhu, Kuan Shen, Qiang Su, Guozhong Yao, Xiaochen Chen

**Affiliations:** ^1^ Department of Gastroenterology, Taizhou Second People’s Hospital Affiliated to Yangzhou University, Taizhou, Jiangsu, China; ^2^ Department of General Surgery, Liyang People’s Hospital, Liyang Branch Hospital of Jiangsu Province Hospital, Liyang, Jiangsu, China; ^3^ Department of General Surgery, the First Affiliated Hospital of Nanjing Medical University, Nanjing, Jiangsu, China; ^4^ The First Clinical Medical College of Guizhou University of Traditional Chinese Medicine, Guiyang, Guizhou, China; ^5^ Department of Radiation Oncology, The Affiliated Suzhou Hospital of Nanjing Medical University, Suzhou Municipal Hospital, Suzhou, Jiangsu, China

**Keywords:** colorectal cancer, immunosenescence, metabolic reprogramming, tumor microenvironment, immunotherapy

## Abstract

Colorectal cancer (CRC), as a highly prevalent malignant tumor worldwide, has a persistently high incidence and mortality rate. In recent years, metabolic reprogramming and immunosenescence have received extensive attention as key mechanisms for tumorigenesis, development and treatment resistance. Metabolic reprogramming not only provides energy and biosynthetic precursors for tumor cells, but also regulates immune responses by reconstructing the tumor microenvironment (TME). Immunosenescence is characterized by the depletion of effector immune cell function and the increase in the proportion of immunosuppressive cells. The two jointly promote the immune escape and therapeutic resistance of CRC. This article systematically reviews the research progress of metabolic reprogramming and immunosenescence in colorectal cancer and explores the related targeted therapeutic strategies, aiming to provide a new theoretical perspective for the precise treatment of CRC.

## 1 Introduction

Colorectal cancer (CRC), as a highly prevalent malignant tumor in the global digestive system, has a persistently high incidence and mortality rate. According to GLOBOCAN 2022 data, there are approximately 1.926 million new cases and 904,000 deaths worldwide each year. The incidence rate ranks third among malignant tumors, and the mortality rate ranks second (Bray et al., 2024).

While early-onset CRC is rising, older adults still represent the majority of CRC cases and deaths. Given the global trend of population aging, the burden of CRC is projected to grow further (He et al., 2025). Age-associated changes such as immunosenescence, chronic low-grade inflammation, gut microbiota dysbiosis, and metabolic dysfunction collectively contribute to tumorigenesis and immune evasion (Jin et al., 2022). These challenges require more precise, personalized, and elderly-friendly screening and treatment strategies. In recent years, the rise of immunotherapy and metabolic-targeted therapies has drawn significant attention to the interaction between metabolic reprogramming and the immune microenvironment in CRC. Immunosenescence—characterized by impaired adaptive immunity and chronic inflammation—weakens antitumor immune surveillance (Yu et al., 2024). Simultaneously, metabolic reprogramming enhances tumor cell survival and immune evasion through modulation of glucose, lipid, and amino acid metabolism (Nenkov et al., 2021; Sun et al., 2024). Together, these processes synergistically shape an immunosuppressive tumor microenvironment, playing a key role in CRC progression and therapy resistance.

Thus, an in-depth exploration of the interplay between immunosenescence and metabolic reprogramming, especially in elderly CRC populations, may offer novel insights for precision oncology. This review aims to comprehensively summarize recent advances in this field and discuss their potential applications in personalized treatment approaches.

## 2 Mechanisms and roles of metabolic reprogramming in colorectal cancer

**FIGURE 1:**
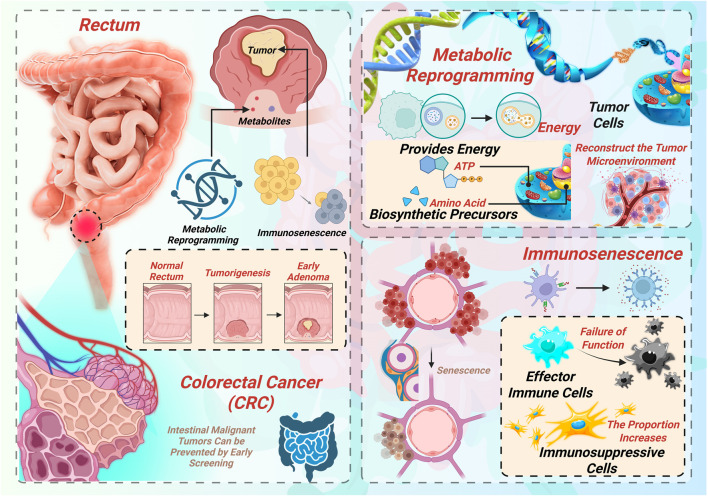
Overview of metabolic reprogramming and immunosenescence in colorectal cancer (CRC).

To better understand CRC progression and therapeutic resistance, it is essential to first elucidate the specific metabolic adaptations of CRC cells and how these alterations shape the tumor microenvironment. Tumor metabolic reprogramming refers to the adaptive shift in cellular metabolism that cancer cells undergo in response to specific microenvironmental stresses—such as hypoxia and nutrient deprivation—to support rapid proliferation, migration, and immune evasion (Qin et al., 2024). CRC cells exhibit a variety of such metabolic adaptations, primarily involving alterations in glucose metabolism, lipid metabolism, amino acid metabolism, and metabolic coupling with the tumor microenvironment. A schematic summary of these processes is illustrated in Figure 1, showing the intertwined roles of metabolic reprogramming and immunosenescence in CRC development.

### 2.1 Aerobic glycolysis (Warburg effect)

CRC cells tend to rely on glycolysis for energy production even in the presence of oxygen—a phenomenon known as aerobic glycolysis or the Warburg effect (Qin et al., 2024). This metabolic shift involves the upregulation of key enzymes such as glucose transporter 1 (GLUT1), hexokinase 2 (HK2), and lactate dehydrogenase A (LDHA), which collectively enhance glucose uptake and lactate production. These processes generate metabolic intermediates that fuel rapid cell proliferation (Hu et al., 2023). Research by Shen et al. demonstrated that elevated expression of HK2 and GLUT1 is associated with poor prognosis in CRC. METTL3 promotes this process by stabilizing HK2 and GLUT1 mRNA through IGF2BPs-mediated m6A modification, thereby enhancing glycolytic activity and CRC cell proliferation, ultimately driving tumor progression (Shen et al., 2020).

### 2.2 Reprogramming of amino acid metabolism

Glutamine is a critical nitrogen and carbon source for CRC cells. Glutamine deficiency has been shown to enhance EMT, promoting CRC recurrence and metastasis (Sun H. et al., 2022). Under high nitrogen demand, CRC cells upregulate glutamine transporters (e.g., SLC1A5) and glutaminase (GLS) to increase glutamine uptake and catabolism. This results in the production of α-ketoglutarate (α-KG) for the TCA cycle, and glutathione for maintaining redox balance and resisting oxidative stress and therapy-induced apoptosis. In addition, the tryptophan–kynurenine pathway (via IDO/TDO-AhR) is frequently activated in CRC (Chang et al., 2024). Its metabolite, kynurenine, exerts potent immunosuppressive effects by inducing T cell exhaustion and expanding regulatory T cells (Tregs), thereby facilitating immune evasion and enhancing tumor cell survival (Zhang et al., 2021).

### 2.3 Metabolic coupling between tumor cells and the microenvironment

CRC cells engage in complex metabolic interactions with components of the tumor microenvironment, including cancer-associated fibroblasts (CAFs), gut microbiota, and immune cells. For instance, CAFs can support tumor metabolism through the “reverse Warburg effect,” providing lactate as an energy source. Additionally, CRC cells absorb fatty acids and phospholipids secreted by CAFs, promoting their migration (Lyu et al., 2024; Gong et al., 2020). Metabolites from the gut microbiota, such as butyrate and secondary bile acids, also act as metabolic bridges linking microbial activity with tumor metabolism and immune regulation. Butyrate, for example, induces apoptosis in CRC cells by inhibiting histone deacetylases (HDACs), whereas an imbalance in secondary bile acids can activate inflammatory pathways like NF-κB, driving tumor proliferation and immune evasion (Qu et al., 2023).

## 3 Characteristics and roles of immunosenescence in colorectal cancer

While tumor metabolic reprogramming significantly influences CRC progression, age-related immune decline—immunosenescence—also profoundly impacts tumor immunity. Thus, understanding the dynamics of immunosenescence provides additional insight into why CRC remains resistant to conventional therapies, especially among elderly patients. Immunosenescence refers to the structural and functional decline of the immune system during physiological aging. It is characterized by reduced immune cell numbers, impaired function, and chronic activation of inflammatory mediators (Wang Y. et al., 2024). As the CRC patient population continues to age, immunosenescence has emerged as a key factor influencing tumor development, progression, and therapeutic response.

### 3.1 Functional decline of T Cell lineages

CD8^+^ cytotoxic T lymphocytes (CTLs) play a central role in antitumor immunity by directly killing malignant cells. High levels of CD8^+^ T cell infiltration—within the tumor core or invasive margin (known as the “Immunoscore”)—have been identified as strong prognostic indicators for recurrence and overall survival in CRC (Mlecnik et al., 2020). With age, the thymus undergoes progressive involution, a hallmark of T cell aging, reducing the output of naïve T cells (Kientega et al., 2024). In CD8^+^ T cells, expression of CD28 is downregulated, while aging markers such as CD57 and KLRG1 are upregulated (Pangrazzi and Weinberger, 2020), leading to terminal differentiation into phenotypes with diminished proliferative and cytotoxic capacities. These senescent T cells exhibit poor responsiveness and are ineffective at clearing tumor cells in CRC. Thymic involution reduces the production of naïve T cells, significantly narrowing the peripheral T cell receptor (TCR) repertoire (Cao et al., 2023). This reduction compromises antigen recognition, limits responses to neoantigens, and weakens immune surveillance against tumors.

In the CRC tumor microenvironment—particularly in elderly patients—there is marked enrichment of regulatory T cells (Tregs). Colonic adenocarcinoma patients show accumulation of PD-1^+^ Tregs, with elevated levels of suppressive molecules such as CTLA-4 and IL-10, which impair the local effector function of CD8^+^ T cells (Mokhta et al., 2023). Meanwhile, markers of exhaustion such as PD-1 and TIM-3 are increasingly expressed on CD8^+^ T cells; blockade of these pathways can partially restore their function (Deng et al., 2010). The expansion of Tregs and accumulation of exhausted CD8^+^ T cells collectively establish an immunosuppressive microenvironment that facilitates CRC immune evasion.

### 3.2 Innate immune decline and inflammaging in CRC

With advancing age, innate immunity in CRC patients undergoes marked functional decline: NK cells from individuals over 70 years exhibit sharply reduced perforin and granzyme B, while downregulated NKG2D expression further weakens their tumour-lytic capacity and predicts poorer postoperative outcomes; dendritic-cell maturation and co-stimulatory signalling (CD80/CD86) likewise diminish, blunting antigen presentation and T-cell priming (Maciejewski et al., 2013). This immunodeficiency is exacerbated by “inflammaging,” a chronic, low-grade inflammatory milieu typified by raised IL-6, TNF-α, C-reactive protein and HMGB1, which drive STAT3-and NF-κB-mediated proliferation, angiogenesis, epithelial–mesenchymal transition and recruitment of suppressive myeloid and regulatory cells, thereby re-shaping the tumour microenvironment toward immune evasion (Li et al., 2024). Consequently, immunosenescent CRC patients respond less favourably and for shorter durations to PD-1/PD-L1 blockade, underscoring the need to stratify individuals by immunosenescence status when optimising immunotherapy regimens (Kim et al., 2022; Verma et al., 2022).

## 4 Interplay between metabolic reprogramming and immunosenescence in colorectal cancer

**FIGURE 2:**
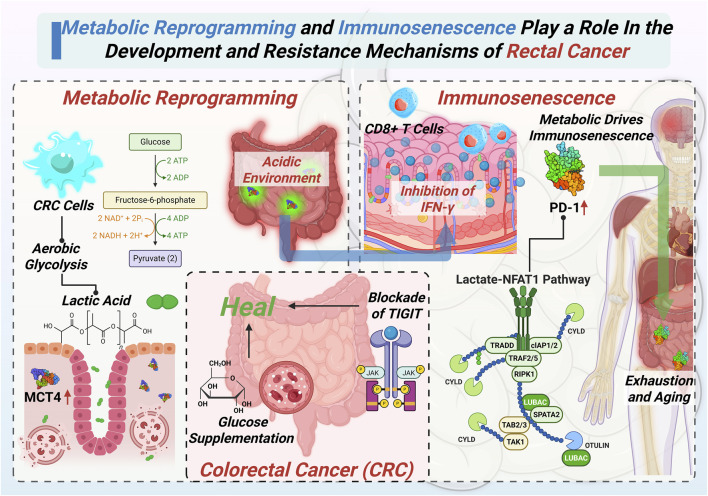
Detailed mechanisms highlighting how metabolic reprogramming and immunosenescence synergistically promote the progression and therapeutic resistance of CRC.

Although metabolic reprogramming and immunosenescence have traditionally been studied independently, recent evidence highlights significant interactions between these two mechanisms. Clarifying their mutual influence is critical to fully grasp the complexity of CRC development and resistance mechanisms. In the development and progression of CRC, metabolic reprogramming of tumor cells and host immunosenescence do not occur independently. Rather, they interact through multiple levels and pathways, synergistically shaping a tumor microenvironment that is both highly immunosuppressive and metabolically supportive. This interplay not only enhances tumor adaptability and invasiveness but also presents a major obstacle to effective therapeutic intervention. The complex feedback loop between tumor metabolism and immune aging is conceptually illustrated in Figure 2, highlighting key regulatory molecules and therapeutic intervention points.

### 4.1 Metabolic reprogramming drives immune dysfunction

Metabolic abnormalities in tumor cells produce byproducts that suppress immune cell function and accelerate immunosenescence through several mechanisms:

CRC cells engage in aerobic glycolysis, producing large amounts of lactate (Siemińska and Lenart, 2025). Monocarboxylate transporter 4 (MCT4), the main transporter for lactate export, is upregulated in various tumors and is associated with poor prognosis in CRC, especially in left-sided tumors (Abe et al., 2019). Lactate accumulation and its export into the TME via MCT4 lead to a drop in local pH (Sun Q. et al., 2022). This acidified environment inhibits CD8^+^ T cell proliferation and effector cytokine (e.g., IFN-γ) production (Brand et al., 2016), and induces PD-1 upregulation via the lactate–NFAT1 pathway, pushing T cells toward an exhausted or senescent phenotype (Kumagai et al., 2022).

In CRC, tumor and immunosuppressive cells (e.g., MDSCs) often overexpress IDO1 (Shi et al., 2022), accelerating tryptophan degradation into kynurenine (Kyn). Kyn, acting as a ligand for the aryl hydrocarbon receptor (AhR), promotes Foxp3^+^ Treg expansion and suppresses CD8^+^ T cell function (Aristin Revilla et al., 2022). AhR also upregulates PD-1 and induces mitochondrial inactivation, driving T cell apoptosis/exhaustion—a hallmark of “metabolically driven immunosenescence” (Zhai et al., 2018).

CRC cells consume glucose and glutamine (Gln) aggressively via GLUT1-mediated glycolysis and glutamine addiction, depleting TME resources (Zhong et al., 2022). This competition suppresses mTOR signaling and IFN-γ production in tumor-infiltrating CD8^+^ T cells (Chang et al., 2015), and Gln deficiency further impairs T cell proliferation and memory formation (Wang B. et al., 2024). Glucose supplementation or TIGIT blockade can partially reverse this (Shao et al., 2021). Thus, CRC cells create a nutrient-deprived TME that induces energy exhaustion and senescence in T cells through “glucose-glutamine dual deprivation.”

### 4.2 Immunosenescence feeds back to enhance tumor metabolism

Aging immune cells also promote tumor metabolic reprogramming via cytokines and secreted factors: Senescent immune or stromal cells secrete pro-inflammatory SASP factors (e.g., IL-6, IL-8, TNF-α, CXCL1), activating STAT3 and NF-κB pathways in tumor cells (Fitsiou et al., 2022). This upregulates metabolic enzymes such as GLUT1, FASN, and GLS, enhancing glycolysis, lipogenesis, and glutamine dependency (De Simone et al., 2015; Han et al., 2016). SASP factors also stabilize hypoxia-inducible factor HIF-1α, amplifying transcriptional programs for metabolic reprogramming and deepening TME immunosuppression (Camporeale et al., 2014).

Senescent cells—including therapy-induced tumor cells, CAFs, and immune cells—secrete increased exosomes into serum and the TME. These vesicles are rich in metabolic regulatory miRNAs/lncRNAs and enzymes (Zhang et al., 2024). Upon uptake by CRC cells, they trigger nuclear translocation of PKM2 and activate STAT3-dependent glycolytic gene transcription, upregulating GLUT1 and LDHA (Wang et al., 2020; Xie et al., 2023).

### 4.3 A self-reinforcing feedback loop

The “metabolism → immunosenescence” and “immunosenescence → metabolic adaptation” processes form a positive feedback loop that perpetuates the hostile tumor microenvironment:

High lactate/kynurenine → T cell exhaustion/senescence → SASP secretion↑ → Enhanced tumor metabolic reprogramming → Stronger immune evasion → Sustained immunosenescence.

This vicious cycle is a key mechanism behind treatment failure and rapid disease progression in advanced CRC. Disrupting this metabolism-immunity feedback loop may represent a critical breakthrough to improve immunotherapy outcomes.

## 5 Therapeutic strategies targeting the metabolism–immunity axis in colorectal cancer

Given this intertwined relationship between tumor metabolism and immunosenescence, developing therapeutic approaches that simultaneously target both pathways represents an innovative and potentially more effective strategy against CRC. CRC progression is increasingly recognized as being fueled by a reciprocal interaction between tumor metabolic reprogramming and immune dysfunction, particularly immunosenescence. These intertwined processes create a TME that favors immune escape and therapeutic resistance. Consequently, there is growing interest in integrated strategies that disrupt aberrant metabolism while revitalizing anti-tumor immunity. Below, we highlight key therapeutic approaches under investigation that aim to remodel the TME, overcome resistance, and enhance treatment outcomes.

### 5.1 Targeting metabolic reprogramming

Metabolic adaptations enable CRC cells to thrive in hypoxic, nutrient-deprived environments while evading immune detection. Glycolysis inhibitors such as 2-deoxyglucose (2-DG), Lonidamine, and PFKFB3 inhibitors have demonstrated the ability to limit glucose utilization, reduce lactate production, and mitigate acidosis in the TME. These effects not only impair tumor proliferation but also enhance T cell viability and activity, thereby improving the response to PD-1/PD-L1 checkpoint blockade. Glutamine metabolism represents another therapeutic vulnerability. The glutaminase inhibitor CB-839 (Telaglenastat), currently in clinical trials for solid tumors, disrupts glutamine-dependent biosynthesis and immune evasion (Varghese et al., 2021). Preclinical studies suggest it can suppress M2 macrophage polarization in the TME, fostering an immunostimulatory milieu (Oyarce et al., 2021).

Glutaminase inhibitors commonly cause nausea, vomiting, fatigue, and appetite loss in clinical trials. By disrupting core metabolic pathways, they may pose long-term risks to organs like the liver and intestines, warranting caution in patients with poor nutrition or metabolic disorders. Additionally, targeting lipid metabolism has shown promise. Inhibitors of FASN and ACC1 block *de novo* lipogenesis, which is critical for membrane formation and oncogenic signaling. Agents such as TVB-2640 (Denifanstat) promote ferroptosis by increasing polyunsaturated fatty acid (PUFA) content, while ND-646 inhibits ACC1/ACC2 dimerization, effectively curbing fatty acid synthesis and tumor growth Inhibitors of FASN and ACC1 suppress fatty acid synthesis, disrupt membrane formation and signaling, and impair metastasis and immune evasion. TVB-2640 (Denifanstat) increases PUFA levels in CRC cells, inducing ferroptosis. ND-646 inhibits ACC1/ACC2 by preventing dimerization, blocking FA synthesis *in vitro* and *in vivo* (Yu et al., 2023). Table 1 provides a systematic overview of the principal targets, mechanisms of action, and latest pre-clinical and clinical advances of the metabolism-targeted therapeutics discussed in this study.

**TABLE 1:** Representative metabolic targeted drugs and their clinical development status.

Drug name	Molecular target	Drug class	Clinical trial status
2-Deoxy-D-glucose (2-DG)	Hexokinase (competitive inhibitor of glucose metabolism)	Glycolysis inhibitor	Phase II (e.g., glioblastoma, prostate cancer)
Lonidamine	MCT1/MCT4 (monocarboxylate transporter inhibitors)	Lactate transport inhibitor	Phase III (multiple solid tumors; mainly historical studies)
PFK-015	PFKFB3 (key glycolytic enzyme)	Glycolysis pathway inhibitor	Preclinical/Early-phase exploratory
KAN0434138	PFKFB3	Highly selective glycolysis inhibitor	Phase I (solid tumors, NCT05245674)
CB-839 (Telaglenastat)	Glutaminase (GLS1)	Glutamine metabolism inhibitor	Phase II/III (RCC, NSCLC, some trials terminated)
TVB-2640 (Denifanstat)	Fatty acid synthase (FASN)	Lipid synthesis inhibitor	Phase III (breast cancer, glioma; FASCINATE-2 ongoing)
ND-646	Acetyl-CoA carboxylase (ACC1/ACC2)	Lipid synthesis inhibitor	Preclinical (validated in animal models)

### 5.2 Reversing immunosenescence

Immune aging is a major barrier to durable anti-tumor immunity in CRC, particularly among older patients. Senolytic agents, such as navitoclax and the dasatinib–quercetin (D + Q) combination, selectively eliminate senescent cells that contribute to immunosuppression (Bogdanova et al., 2024). Navitoclax (ABT-263) induces apoptosis in BCL-2-dependent senescent cells and activates STING signaling, leading to enhanced type I interferon production and T cell recruitment (Zhang et al., 2025). As a typical senolytic drug, its dose-limiting toxicity is mainly manifested as thrombocytopenia, because BCL-xL inhibition affects platelet survival. This side effect is more risky for elderly patients (those with impaired bone marrow hematopoietic function) and patients who need combined chemotherapy or radiotherapy. In contrast, senomorphic agents aim to suppress the pro-inflammatory SASP without killing senescent cells. mTOR inhibitors like rapamycin and metabolic regulators such as metformin reduce SASP production, preserve T cell homeostasis, and delay immunosenescence (Zhou et al., 2025). Efforts to rejuvenate T cell function directly are also underway. Cytokines such as IL-7 and IL-15 enhance the function of aged T cells, while adoptive cell therapies—including CAR-T, TCR-engineered T cells, and young donor-derived T cells—offer strategies to restore effective cytotoxicity in immunosenescent patients (Hombach et al., 2020). Table 2 presents a concise summary of the key strategies, potential clinical value, and recent research progress of the immunological and senescence-modulating therapies explored in this work.

**TABLE 2:** Immunomodulator and aging-targeted therapies in clinical investigation.

Drug/combination name	Molecular target	Drug class	Clinical trial status
Navitoclax (ABT-263)	BCL-2/BCL-xL	BH3 mimetic (pro-apoptotic agent)	Phase II (myelofibrosis, leukemia)
ABT-199 (Venetoclax)	Selective BCL-2 inhibitor	BH3 mimetic	Marketed (hematologic malignancies); Phase II (solid tumors)
Dasatinib + Quercetin (D + Q)	Senescence pathways (p53/p21, BCL-2 family)	Senolytics (senescent cell clearance agents)	Phase II (diabetic nephropathy, pulmonary fibrosis, aging-related diseases)
Sirolimus (Rapamycin)	mTORC1	mTOR inhibitor	Marketed (organ transplantation); Phase II (anti-aging studies, e.g., canine longevity)
Metformin	AMPK activator/Mitochondrial complex I inhibitor	Insulin sensitizer	Marketed (diabetes); Phase III (anti-aging TAME trial)
IL-7/IL-15	T cell proliferation and survival factors	Immunomodulatory cytokines	Phase I/II (cancer immunotherapy, HIV immune reconstitution)

### 5.3 Synergistic combination therapies

Combination strategies that integrate metabolic inhibition with immune checkpoint blockade and senescence-targeted therapies are showing synergistic effects in preclinical models. For instance, combining 2-DG with anti-PD-1 antibodies under a fasting-mimicking diet in CT26 CRC mouse models reduced intratumoral lactate levels and enhanced CD8^+^ T cell activation, resulting in significant tumor regression and prolonged survival. Although promising, these results await clinical validation (Peng et al., 2025). Similarly, CB-839 in combination with nivolumab has entered early-phase trials for solid tumors (Raczka and Reynolds, 2019). While CRC-specific data are limited, studies in melanoma models suggest this combination promotes T cell infiltration and augments checkpoint inhibitor efficacy (Varghese et al., 2021). In models of therapy-induced senescence (TIS), immune checkpoint inhibitors alone have limited efficacy due to the accumulation of immunosuppressive senescent cells. The addition of senolytics like ABT-263 restored immune competence by clearing these cells and enhancing the anti-tumor effects of PD-L1 blockade (Maggiorani et al., 2024).

### 5.4 Prospects for personalized therapy

The future of CRC therapy lies in personalized, multi-dimensional interventions that account for both metabolic and immune states. Advances in single-cell RNA sequencing, spatial transcriptomics, and artificial intelligence are enabling refined patient stratification based on tumor-intrinsic and TME features. Emerging biomarker panels now allow stratification by metabolic and immune profiles. Together, these approaches represent a shift from monotherapy to rational, biomarker-driven combination therapies that modulate both tumor metabolism and immune function, offering new avenues to overcome resistance and improve survival in CRC patients.

Despite these exciting developments, several real-world limitations may hinder widespread clinical adoption. Technologies such as single-cell RNA sequencing and spatial transcriptomics remain costly and require access to specialized platforms and significant bioinformatics expertise—resources that may not be available in many clinical settings. Additionally, variability in sample quality, processing standards, and data interpretation presents further challenges to integration into routine workflows. Addressing these barriers through continued technological innovation, training, and infrastructure development will be essential to fully realize the potential of personalized CRC therapy.

## 6 Conclusion and outlook

CRC, as one of the most prevalent and lethal malignancies globally, presents formidable challenges due to its intricate pathogenesis and high therapeutic resistance. Emerging evidence highlights the central roles of metabolic reprogramming and immunosenescence not only as independent contributors to tumor progression but also as interactive forces that establish a profoundly immunosuppressive and metabolically adaptive TME. This dynamic “metabolism–immunity feedback loop” is now increasingly recognized as a key driver of CRC progression and a critical barrier to effective immunotherapy, especially in elderly patients.

In this pathological loop, aberrant tumor metabolism ensures a steady supply of energy and biosynthetic substrates while generating immunosuppressive byproducts—such as lactate and kynurenine—that impair T cell function and accelerate immune aging. Simultaneously, senescent immune cells amplify tumor metabolic adaptation through the secretion of SASP factors, which promote tumor plasticity and immune evasion. This mutually reinforcing mechanism underpins the limited efficacy of immunotherapy and targeted treatments in aging CRC populations and suggests that both metabolic and immune dysregulation must be addressed in tandem.

Looking forward, future research should focus on constructing a comprehensive map of the CRC metabolism–immunity landscape using single-cell and spatial multi-omics tools, enabling the identification of actionable regulatory nodes. Parallel efforts should aim to develop multi-targeted interventions that simultaneously address key mechanisms such as lactate accumulation, glutamine addiction, and T cell exhaustion. Personalized therapeutic strategies—tailored to patient age, immunosenescence profiles, and metabolic states—may offer greater precision and efficacy. Importantly, real-world clinical trials that incorporate diverse patient populations are needed to evaluate the safety and effectiveness of these integrated approaches.

In conclusion, deeper mechanistic insight into the interplay between metabolic reprogramming and immunosenescence offers an opportunity to overcome longstanding barriers in CRC therapy. Disrupting this pathological feedback loop through precise, personalized, and age-adapted interventions holds promise for transforming CRC management and improving outcomes in patient populations most vulnerable to treatment failure.
